# Influence of Donor Obesity on Adipose-Derived Stem Cell Function and Therapeutic Efficacy

**DOI:** 10.3390/cells15100946

**Published:** 2026-05-21

**Authors:** Marva Khalid, Marvin L. Frommer, Jeries Abu-Hanna, Benjamin J. Langridge, Clara Calero Pages, Laura Awad, Peter E. M. Butler

**Affiliations:** 1 Charles Wolfson Centre for Reconstructive Surgery, Royal Free Hospital, London NW3 2QG, UK; marva.khalid.25@ucl.ac.uk (M.K.); benjamin.langridge.14@ucl.ac.uk (B.J.L.);; 2 Department of Surgical Biotechnology, Division of Surgery & Interventional Science, University College London, London NW3 2PS, UK; 3 Division of Medical Sciences, University of Oxford, Oxford OX3 9DU, UK; 4 Department of Plastic Surgery, Imperial College Healthcare NHS Trust, London W2 1NY, UK; 5 Department of Plastic Surgery, Royal Free Hospital, London NW3 2QG, UK

**Keywords:** adipose-derived stem cells, obesity, subcutaneous adipose tissue, cellular senescence, mitochondrial dysfunction, epigenetic memory, secretome, adipogenesis

## Abstract

Adipose-derived stem cells (ADSCs) are widely used in regenerative medicine and are considered key effectors underlying the therapeutic efficacy of autologous fat grafting for scarring and skin fibrosis, yet clinical outcomes remain variable. This review examines how obesity alters the adipose microenvironment through chronic inflammation and metabolic dysfunction, resulting in epigenetic changes, mitochondrial impairment, oxidative stress, and premature cellular senescence in ADSCs. ADSCs from obese individuals exhibit reduced stemness, impaired differentiation, and a pro-inflammatory secretome with diminished regenerative capacity. While weight loss may partially reverse these effects, persistent epigenetic and functional memory limits full recovery. This review argues that donor metabolic status is a determinant of ADSC therapeutic potency and discusses key challenges and opportunities for improving regenerative outcomes.

## 1. Introduction

Adipose-derived stem cells (ADSCs) are used widely throughout regenerative medicine, both pre-clinically in tissue engineering and clinically via autologous fat transfer [1]. They are easily harvested from subcutaneous discarded fat or via liposuction. ADSCs, as mesenchymal stem cells, have potent paracrine activity and a capacity to differentiate along adipogenic, osteogenic, chondrogenic, myogenic, and neurogenic lineages [2,3,4,5,6]. Adipose tissue is compartmentalised into distinct depots, with subcutaneous and visceral fat exhibiting divergent cellular and metabolic characteristics [7,8]. This review focuses on subcutaneous adipose-derived stem cells, which constitute the primary harvest source for autologous fat transfer and other clinical regenerative applications [1,3]. In this review, ADSCs refers to adipose-resident mesenchymal progenitor cells with stem-like properties that have not yet undergone full preadipocyte commitment. This population is heterogeneous and includes cells across a spectrum of adipogenic commitment, with distinct functional states and subpopulations that are variably defined across the literature [9,10].

Autologous fat transfer (AFT) is widely used to correct tissue volume loss, but it also improves surrounding tissue quality. Studies show AFT promotes collagen remodelling, increases blood supply, and reduces markers of fibrosis, dermal thickness, and scar size [3,5]. These tissue-level effects translate into measurable clinical benefit across fibrotic conditions. In head and neck oncology patients with radiation-induced fibrosis and volumetric defects, lipotransfer produced functional and aesthetic improvement in 97% of patients, with significant improvement in psychological outcomes [11]. In systemic sclerosis, stem cell-enriched lipotransfer produced significant improvements in oro-facial function in 62 patients, with all validated psychological outcomes improving concurrently [12]. AFT has similarly been applied to lichen sclerosus, Dupuytren’s disease, and scar-related contractures, with clinical studies reporting improved function and pain across these conditions [5,13]. Fat grafting has also been applied to chronic non-healing wounds, where stromal vascular fraction and ADSCs may contribute to tissue repair [1]. Across these applications, the volumetric effect of the graft alone cannot account for the observed improvements [5].

The mechanisms underlying the anti-fibrotic effects of AFT remain incompletely understood, though ADSCs within the grafted tissue are proposed as key mediators [12]. Adipose tissue is highly regenerative and contains a diverse mix of cells within the stromal vascular fraction (SVF), including immune cells, endothelial cells, and adipose-derived stem cells (ADSCs). Although ADSCs represent a small proportion of grafted cells, they are thought to play a central role in mediating these anti-fibrotic effects [5].

Obesity fundamentally remodels the adipose microenvironment through chronic low-grade inflammation, insulin resistance, and endocrine dysfunction [8,14]. While surgical techniques for fat harvesting have become increasingly standardised, persistent variability in clinical outcomes points to the important role of intrinsic patient or disease characteristics [15]. Because ADSCs reside within an altered niche, systemic conditions like obesity can modify their baseline phenotype and functional capacity [7,15,16]. Evidence suggests that ADSCs harvested from obese donors exhibit impaired proliferation, differentiation, premature senescence, and reduced paracrine potency compared to cells from lean individuals [16,17]. If these changes translate clinically, obesity could compromise both fat grafting outcomes and pre-clinical research. This review examines the current evidence on how obesity alters ADSC biology and evaluates the implications for regenerative medicine and clinical practice.

## 2. The Effect of Obesity on Subcutaneous Fat

Adipose tissue functions as a highly dynamic endocrine and metabolic organ responsible for energy storage and homeostasis [6]. The stromal vascular fraction (SVF) of this tissue houses a heterogeneous cell population [18]. Recent single-cell and flow cytometric analyses reveal that this compartment comprises ADSCs, preadipocytes, endothelial cells, fibroblasts, and a diverse immune cell population including macrophages, mast cells, neutrophils, and T lymphocytes [9,19,20]. Under physiological conditions, adipose progenitor populations, including ADSCs, preadipocytes, and intermediate states of adipogenic commitment, constitute a major non-adipocyte compartment within the stromal vascular fraction, representing approximately 40% of cells in integrated single-cell analyses [9,20]. Together, these progenitors support healthy adipose remodelling and regenerative capacity [9,20]. Through these progenitors, subcutaneous adipose tissue can appropriately store excess fat via the recruitment of new cells (hyperplastic expansion) [21]. During periods of chronic positive energy balance, the subcutaneous fat depot undergoes pathological remodelling. Adipocytes initially experience hypertrophic expansion to store the lipid surplus, eventually reaching a physical threshold that triggers localised tissue hypoxia and mechanical stress [22]. Hypertrophic adipocytes begin to undergo cell death, leading to the formation of crown-like structures that disrupt the local tissue microarchitecture [23]. The extracellular matrix concurrently experiences pathological remodelling, resulting in dense pericellular fibrosis [24].

Obesity promotes immune cell accumulation in adipose tissue at the expense of the resident progenitor pool. Hypertrophic adipocytes and surrounding stromal cells increase their secretion of pro-inflammatory cytokines, recruiting circulating monocytes that subsequently polarise into classically activated M1 macrophages [19]. Myeloid immune cells accumulate progressively with increasing body mass index (BMI) in subcutaneous adipose tissue [25], and pro-inflammatory CD11c+ macrophages displace the anti-inflammatory M2-polarised macrophages that typically predominate in lean tissue [20,26]. This immunological shift is reflected in the upregulated expression of cytokines such as interleukin-1 beta (IL-1β) by obese mesenchymal stromal cells (MSCs) [27], and a concurrent reduction in circulating adiponectin, an anti-inflammatory adipokine whose loss amplifies the pro-inflammatory milieu [23]. Beyond immune polarisation, obesity induces broad remodelling of the stromal vascular niche, encompassing myeloid accumulation and enrichment of pro-inflammatory immune-cell subsets [10,25]. These converging alterations collectively degrade the microenvironmental conditions required for progenitor maintenance and function. Within this inflamed and fibrotic niche, the resident progenitor pool undergoes depletion and dysregulation, with obesity altering not only ADSC abundance but also their differentiation state. Flow cytometric analysis demonstrates that CD90+ progenitor-enriched cells are reduced in morbidly obese individuals compared to lean controls (19.11% vs. 35.31%; *p* < 0.05) [28]. Single-cell profiling demonstrates that while ADSCs increase with BMI, more committed preadipocyte/adipocyte populations decrease, suggesting expansion of an immature or developmentally arrested ADSC pool rather than effective adipogenic maturation [10,25]. Subtype-level and integrated single-cell and spatial analyses have further identified metabolically distinct ADSC populations whose distribution associates with type 2 diabetes, insulin resistance, adipocyte morphology, and impaired lipid mobilisation [9,29]. These two shifts are not independent of the inflammatory microenvironment: sustained macrophage activation within the obese niche directly impairs adipogenesis and promotes fibrosis, further eroding the regenerative capacity of the residual progenitor pool [30]. Collectively, these findings indicate that obesity alters both ADSC function and ADSC population dynamics, creating a progenitor-rich but functionally impaired SVF. The specific intrinsic defects acquired by these progenitors upon exposure to the obese microenvironment are considered across six interconnected functional domains: epigenetic and genomic scarring; mitochondrial dysfunction and oxidative stress; premature cellular senescence and loss of stemness; impaired adipogenesis and pro-fibrotic reprogramming; a dysregulated pro-inflammatory secretome with diminished immunomodulatory capacity; and impaired angiogenic capacity.

## 3. Epigenetic and Genomic Scarring

The obesogenic microenvironment compromises ADSCs through a coordinated epigenetic triad: stable 5-methylcytosine (5mC) DNA methylation, dynamic 5-hydroxymethylcytosine (5hmC) remodelling, and long non-coding RNA dysregulation.

### 3.1. Epigenetic Memory and Alterations in DNA Methylation

At the core of the altered ADSC phenotype are global 5mC modifications, which permanently alter early lineage commitment and cause apoptotic resistance. Hundreds of specific DNA loci exhibit altered methylation patterns that distinguish subcutaneous ADSCs of lean from obese donors [31]. Promoter hypomethylation upregulates the developmental gene *TBX15* in obese ADSCs, driving mitochondrial dysfunction in mature adipocytes [31]. The anti-apoptotic *Survivin* gene undergoes promoter hypermethylation and consequent reduction in mRNA expression in obesity; paradoxically, however, survivin protein levels remain elevated in obese ADSCs. Reduced microRNA-203 expression and impaired autophagy-dependent degradation sustain survivin protein accumulation. M1 macrophage-derived IL-1β signalling contributes to its upregulation, though elevated survivin persists ex vivo in the absence of ongoing inflammatory stimuli, implicating the post-transcriptional and post-translational mechanisms as the more durable determinants. Together, these mechanisms lead to survivin accumulation, conferring abnormal apoptotic resistance upon obese ADSCs, accompanied by reciprocal suppression of p53 [32].

### 3.2. DNA Hydroxymethylation Shifts

Unlike stable 5mC, 5hmC acts as a highly responsive epigenetic modifier that is remodelled by obesity. This remodelling is mediated by Ten-Eleven Translocation (TET) enzymes, which catalyse the oxidative conversion of 5mC to 5hmC and directly regulate the expression of nuclear-encoded mitochondrial genes [33]. Locus-specific 5hmC shifts imprint a molecular scar on genes controlling apoptosis, senescence, and proliferation [14,33], simultaneously driving metabolic, autophagic, and inflammatory deterioration in ADSCs. Metabolically, aberrant 5hmC tagging of genes essential for ATP synthesis and fatty acid metabolism directly impairs cellular energetics [33]. These epigenetic shifts disrupt cellular recycling by altering autophagy-related genes such as *STX12* and *SLC25A4*, accelerating early autophagosome consumption and reducing overall autophagic utilisation efficiency [34]. This dynamic 5hmC rewiring activates cytokine production pathways, triggering an overproduction of IL-1β that limits the anti-inflammatory capacity of subcutaneous ADSCs [27]. Aberrant 5hmC marks on apoptosis and senescence-related genes were partially reversed by vitamin C, a TET enzyme co-factor, providing preliminary evidence that this hydroxymethylation landscape may represent a tractable epigenetic target in obese ADSCs [14].

### 3.3. Long Non-Coding RNA Dysregulation

Linking DNA-level modifications to transcriptomic output, long non-coding RNAs (lncRNAs) emerge as key regulators in the obese ADSC epigenome. Upstream promoter alterations drive specific lncRNAs to impair normal adipogenesis and precipitate premature cellular senescence. For example, promoter hypomethylation significantly upregulates the lncRNA *PANDAR* in ADSCs from obese and diabetic subjects, enhancing its p53-mediated induction to accelerate senescence and induce cell cycle arrest [35]. Beyond senescence, obesity disrupts an essential adipogenic lncRNA network, evidenced by the dysregulation of six specific transcripts [36]. Additionally, obesity upregulates the lncRNA *HOXA11-AS1*, demonstrating how the obesogenic environment alters the non-coding regulatory networks that govern lipid-related gene transcription and healthy fat expansion [37]. The principal epigenetic alterations identified across these three regulatory layers are summarised in Table 1.

## 4. Mitochondrial Dysfunction and Oxidative Stress

### 4.1. Structural Alteration of Mitochondria

Driven by these epigenetic and genomic alterations, obesity triggers the physical swelling and structural degradation of mitochondria in ADSCs [33]. This damage manifests as a reduction in both overall mitochondrial mass and the mitochondrial DNA (mtDNA) copy number [38]. The remaining mitochondria appear abnormally thinned and disordered, featuring distinct swollen regions devoid of functional cristae [38].

### 4.2. Functional Alteration and Metabolic Shift

Consequently, this structural degradation compromises the oxidative phosphorylation pathway [38], precipitating a decline in both ATP synthesis and mitochondrial membrane potential [33]. ADSCs display a decreased oxygen consumption rate as they exhibit impaired glucose oxidation and rely on fatty acid oxidation to survive [38,39]. In response to this impaired oxidative phosphorylation, ADSCs from elderly and obese individuals exhibit a compensatory upregulation of aerobic glycolysis and enhanced glucose conversion to glycogen [40]. This intracellular glycogen accumulation downregulates *SIRT1* and *SIRT6* metabolic sensors [40]. In parallel, obesity impairs lysosomal function in ADSCs, disrupting the autophagic process by which damaged organelles are delivered to and degraded within lysosomes. This impairment prevents the selective removal of damaged mitochondria through mitophagy, causing their progressive accumulation within the cell [38].

As a result, impaired mitophagy permits unchecked reactive oxygen species (ROS) production [38], driving cellular oxidative stress characterised by elevated intracellular superoxide production [16]. This pro-oxidant shift is exacerbated by a marked overexpression of NADPH oxidases in subcutaneous ADSCs from obese subjects with metabolic syndrome (MetS) [41]. Compounding this burden, a significant downregulation of the enzymes responsible for generating cytosolic acetyl-CoA, namely *ACLY* and *ACSS2*, alongside downregulation of malonyl-CoA decarboxylase (MCD), impairs the endogenous antioxidant capacity of these ADSCs through two converging mechanisms. First, reduced acetyl-CoA availability disrupts histone acetylation, altering the transcription of protective cellular proteins. Second, the failure of MCD to convert malonyl-CoA into acetyl-CoA causes excessive malonyl-CoA accumulation, promoting aberrant protein malonylation. These disturbances suppress antioxidant enzyme expression, including catalase and *SOD3*, and are associated with a concurrent reduction in the antioxidant transcription factor *FOXO1* [42].

## 5. Premature Cellular Senescence and Decrease in Stemness

### 5.1. Senescence and Subcellular Defects

The obese adipose tissue microenvironment compromises subcutaneous ADSCs by causing premature cellular senescence [16]. This transition is accompanied by mitochondrial and lysosomal dysfunction, as well as shortening of the primary cilium, which impairs the cells’ capacity to respond to extracellular stimuli [38,43]. Consequently, the senescent phenotype manifests through the upregulation of markers such as p16, p53 [14,16,21], and the senescence-associated β-galactosidase (SA-β-gal) gene (*GLB1*) [16,21].

### 5.2. Autophagy and Apoptosis Dysregulation

In obese ADSCs, autophagy is impaired by the dysregulation of genes such as *STX12* and *SLC25A4* [34]. Concurrently, local pro-inflammatory cytokines such as IL-1β [27,32] drive high levels of the anti-apoptotic protein Survivin [32]. This overexpression shields obese ADSCs from stress-induced apoptosis (e.g., hypoxia and elevated leptin), preventing their clearance and resulting in their accumulation [32]. This apoptotic resistance, coupled with autophagic dysfunction, maintains the cells in their senescent state [34].

### 5.3. Decrease in Stemness and Functional Change

**Figure 1:**
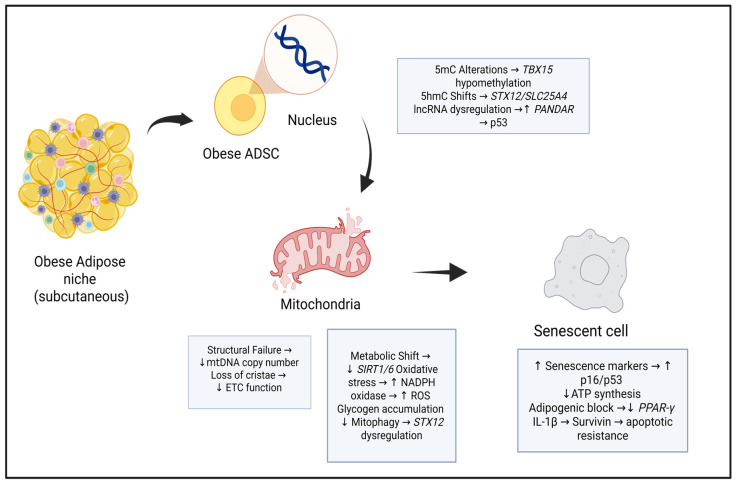
Cellular and molecular changes in obese subcutaneous adipose-derived stem cells (ADSCs). The obese subcutaneous adipose niche drives ADSCs toward a senescent phenotype through converging epigenetic and mitochondrial alterations. Nuclear changes comprise an “epigenetic triad” of 5mC, 5hmC, and lncRNA dysregulation. Concurrent mitochondrial structural failure and metabolic reprogramming compound these effects, culminating in upregulation of senescence markers, impaired adipogenic differentiation, and apoptotic resistance. ↑ indicates an increase or upregulation; ↓ indicates a decrease or downregulation. (Abbreviations: 5hmC, 5-hydroxymethylcytosine; 5mC, 5-methylcytosine; ADSC, adipose-derived stem cell; ATP, adenosine triphosphate; ETC, electron transport chain; IL-1β, interleukin-1 beta; lncRNA, long non-coding RNA; mtDNA, mitochondrial DNA; NADPH, nicotinamide adenine dinucleotide phosphate; *PANDAR*, promoter of CDKN1A antisense DNA damage-activated RNA; PPAR-γ, peroxisome proliferator-activated receptor gamma; ROS, reactive oxygen species; *SIRT1/6*, sirtuin 1/6; *SLC25A4*, solute carrier family 25 member 4; *STX12*, syntaxin 12; *TBX15*, T-box transcription factor 15. Created with BioRender.com).

Once senescent, ADSCs experience a reduction in intrinsic multipotency, evidenced by downregulation of key pluripotent genes such as *OCT4*, *SOX2*, and *NANOG* [44]. Rather than maintaining an uncommitted progenitor state, senescent ADSCs undergo a transcriptomic shift toward a pre-committed, adipocyte-like phenotype [45]. Experimental evidence confirms that this interconnected loss of stemness is linked to altered telomerase activity and diminished DNA telomere length, which restricts self-renewal and contributes to a decrease in proliferative capacity [16]. Furthermore, dysregulated motility impairs their capacity to migrate and invade injury sites for effective tissue repair [46]. The convergence of these epigenetic, mitochondrial, and senescence-associated alterations in obese subcutaneous ADSCs is illustrated in Figure 1.

## 6. Impaired Adipogenesis and Pro-Fibrotic Reprogramming

### 6.1. Impaired Adipogenesis

During healthy adipose tissue expansion, ADSCs proliferate and differentiate to generate new mature adipocytes [47]. Subcutaneous preadipocytes from severely obese patients show reduced intracellular lipid accumulation compared to lean controls [48]. This arrested adipogenesis is associated with microenvironmental triggers, such as tumour necrosis factor-alpha (TNF-α), and elevated expression of mitogen-activated protein 4 kinase 4 (*MAP4K4*), which has been suggested as a potential inhibitor of PPAR-γ activation and adipocyte maturation [49]. Under normal conditions, *ZNF521* maintains mesenchymal progenitor cells in a proliferative, uncommitted state. However, its persistent expression in poorly differentiating cells from individuals with hypertrophic obesity reflects senescence-driven failure to differentiate rather than active maintenance of an uncommitted phenotype [21].

### 6.2. Pro-Fibrotic Reprogramming

In obese individuals without metabolic syndrome, subcutaneous ADSCs appear to resist fibrosis through elevated surface levels of Fibroblast Growth Factor 2 (FGF2) and the TGF-β signalling inhibitor SMAD7, which are associated with decreased *COL1A1* expression, suggesting they may act together to protect against scarring [24]. However, in patients with metabolic syndrome, this protection is lost. These ADSCs concurrently downregulate core adipogenic genes (*SLC2A4* and *PLIN1*), and upregulate fibrotic extracellular matrix proteins (*COL1A1*, *FN1*) [24], ultimately contributing to the pericellular scarring and tissue rigidity characteristic of obesity [24,46].

In obesity, microenvironmental triggers drive the disassembly of the primary cilium, resulting in shortened, defective sensory organelles in human subcutaneous ADSCs [44,46]. This structural impairment disrupts critical communication pathways, such as Hedgehog (Hh) signalling [44], operating in conjunction with the excessive collagen accumulation and matrix rigidity in obese adipose tissue [46].

## 7. Pro-Inflammatory Secretome and Impaired Immunomodulatory and Angiogenic Capacity

### 7.1. The Shift Towards a Pro-Inflammatory Secretome

Driven by previously established premature senescence [16] and metabolic reprogramming [40], obese ADSCs acquire a Senescence-Associated Secretory Phenotype (SASP) [16]. Specifically, the downregulation of *SIRT1* and *SIRT6* correlates with decreased secretion of reparative factors (e.g., TGF-β1) [8] and elevated secretion of pro-inflammatory cytokines (TNF-α, IL-6) and chemokines (MCP-1) [40]. This pro-inflammatory shift is driven by acquired weight rather than genetics, demonstrated by significantly higher *TNF-α* expression in ADSCs from the heavier siblings of weight-discordant monozygotic twins compared to their leaner co-twins [50]. However, ADSCs from the heavier twins also demonstrated greater in vitro immunosuppressive capacity than those from their leaner co-twins, a finding that complicates a straightforward interpretation of obesity-related immunomodulatory impairment [50].

### 7.2. Impaired Immunomodulatory Capacity

The pro-inflammatory secretome impairs immunomodulatory function, as elevated TNF-α [51] and reduced TGF-β1 [8] disrupt macrophage polarisation and T-cell suppression. This is supported by conditioned medium [8] and indirect transwell co-culture experiments [51,52], demonstrating that secreted factors alone are sufficient to drive these defects. In xenogeneic models, human obese ADSCs fail to induce M2 macrophage polarisation and instead promote a pro-inflammatory M1 phenotype in murine macrophages and microglia [52]. This was characterised by nitric oxide activation [52], alongside impaired phagocytic capacity and reduced migratory activity in target macrophages [51]. This polarisation failure was demonstrated in vivo, where transplanted obese human ADSCs failed to resolve tissue inflammation in murine kidneys [51]. This failure extends to adaptive immunity, as obese ADSCs fail to suppress T-cell and B-cell proliferation [8]. The immunomodulatory dysfunction was mechanistically attributed to IL-1β signalling and was partially reversible following treatment with IL-1 receptor antagonist and TGF-β [8]. Furthermore, increasing donor BMI correlates with an inability to decrease IL-2 production in CD4+ T-cells, occurring against a pro-inflammatory secretory background characterised by elevated IL-6 and PGE2 [53]. Despite this attenuated suppression, the capacity to induce regulatory T-cells (CD4+CD25+FOXP3+) remained comparable to that of lean ADSC donors, indicating a selective rather than global loss of immunomodulatory function [53]. Additionally, the CD40-CD40L axis is significantly upregulated in the obese adipose microenvironment; activated CD4+ T-cells expressing CD40L bind to CD40 on ADSCs, triggering NF-κB-mediated IL-8 production and driving subsequent neutrophil recruitment [54]. These broad immunomodulatory defects align with an altered surface immunophenotype featuring upregulated HLA-II and CD106 (VCAM-1), and downregulated CD29 [55].

### 7.3. Impaired Angiogenesis

ADSCs stimulate angiogenesis through the paracrine secretion of growth factors, cytokines, and extracellular vesicles that regulate endothelial cell proliferation, migration and tube formation [56]. They may also differentiate directly into endothelial-like cells, contributing to new microvessel formation [56]. Obesity impairs the pro-angiogenic and vascular repair capacity of subcutaneous ADSCs [16,45,57]. In a study of weight-discordant monozygotic twins, Juntunen et al. isolated the effects of acquired adiposity from genetic background on ADSC angiogenic function. ADSCs from heavier twins exhibited an impaired ability to support endothelial network formation, with leaner co-twins’ ADSCs facilitating 21% larger vasculature area and 25% longer vasculature-like structures [50]. This functional deficit was accompanied by reduced CD146 expression, a pericyte marker central to angiogenic support and endothelial layer stabilisation, suggesting a concurrent loss of pro-angiogenic cellular identity [50]. Obese ADSCs exhibit reduced intrinsic capacity to form capillary-like tubules, with those from subjects with metabolic syndrome showing a statistically significant reduction [41]. This defect extends to an impaired response to pro-angiogenic stimuli such as PMA, accompanied by impaired migration and invasion responses to chemotactic stimuli such as MCP-1 [58]. Consequently, this population loses both the intrinsic and paracrine competency required to support endothelial networks [16].

**Figure 2:**
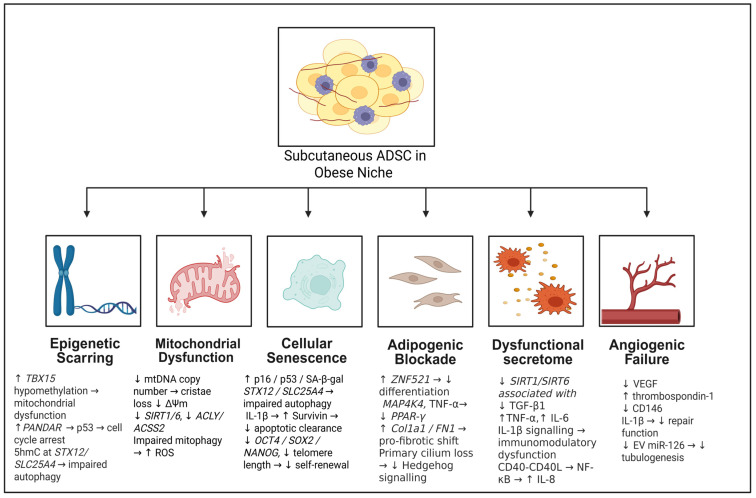
Summary of alterations in subcutaneous adipose-derived stem cells (ADSCs) during obesity. Obesity induces a multifaceted decline in ADSC function driven by six primary hallmarks: epigenetic scarring, mitochondrial dysfunction, cellular senescence, adipogenic blockade, a dysfunctional secretome, and angiogenic failure. The MAP4K4-mediated suppression of PPAR-γ represents a proposed rather than established mechanism. NADPH oxidase upregulation and pro-fibrotic reprogramming (↑ *COL1A1*, ↑ *FN1*) are specific to ADSCs from donors with metabolic syndrome. Survivin protein accumulation occurs despite promoter hypermethylation, consequent to IL-1β signalling and reduced *miR-203* expression. ↑ indicates an increase or upregulation; ↓ indicates a decrease or downregulation. (Abbreviations: 5hmC, 5-hydroxymethylcytosine; *ACLY*, ATP-citrate lyase; *ACSS2*, acyl-CoA synthetase short-chain family member 2; ADSC, adipose-derived stem cell; CD146, cluster of differentiation 146; *COL1A1*, collagen type I alpha 1; EV, extracellular vesicle; *FN1*, fibronectin 1; IL-1β, interleukin 1 beta; IL-6, interleukin 6; IL-8, interleukin 8; *MAP4K4*, mitogen-activated protein 4 kinase 4; MetS, metabolic syndrome; miR, microRNA; mtDNA, mitochondrial DNA; NADPH, nicotinamide adenine dinucleotide phosphate; *NANOG*, Nanog homeobox; NF-κB, nuclear factor kappa B; *OCT4*, octamer-binding transcription factor 4; p16, cyclin-dependent kinase inhibitor 2A; p53, tumour protein 53; PPAR-γ, peroxisome proliferator-activated receptor gamma; ROS, reactive oxygen species; SA-β-gal, senescence-associated beta-galactosidase; *SIRT1/6*, Sirtuin 1/6; *SLC25A4*, solute carrier family 25 member 4; *SOX2*, SRY-box transcription factor 2; *STX12*, Syntaxin 12; *TBX15*, T-box transcription factor 15; TGF-β1, transforming growth factor beta 1; TNF-α, tumour necrosis factor alpha; VEGF, vascular endothelial growth factor; *ZNF521*, zinc finger protein 521; ΔΨm, mitochondrial membrane potential. Created with BioRender.com).

This angiogenic failure is mechanistically driven by a shift in the ADSC secretome [45]. During adipogenic differentiation, obese subcutaneous ADSCs secrete significantly less vascular endothelial growth factor (VEGF) and pro-angiogenic adiponectin, while paradoxically upregulating hepatocyte growth factor (*HGF*) [19,41]. These ADSCs also overexpress the pro-inflammatory cytokine IL-1β, which reduces their repair functionality [27]. This compromised secretome is characterised by elevated *THBS1* mRNA expression and thrombospondin-1 (TSP-1) protein secretion, which directly correlates with donor BMI and reduces tissue repair [28]. Functionally, conditioned medium derived from obese ADSCs shows reduced tubule formation rates in human umbilical vein endothelial cells (HUVECs) [28], and the cells fail to restore *VEGF* expression or stimulate tubulogenesis when co-cultured with injured HUVECs [16]. The primary hallmarks of ADSC dysfunction in obesity are summarised in Figure 2. The underlying pathophysiological mechanisms contributing to each hallmark are detailed in Table 2.

The paracrine deficiency extends beyond soluble factors to ADSC-derived EVs, which show a reduction in pro-angiogenic factors, including VEGF, MMP-2, and microRNA-126, decreasing their capacity to cause endothelial migration or tube formation [17]. In vivo administration of EVs from obese subcutaneous ADSCs fails to preserve myocardial capillary density, consistent with diminished therapeutic repair potency [57]. Beyond its paracrine consequences, obesity disrupts epigenetic programming required for ADSCs to transition into endothelial-like cells. When induced toward an in vitro endothelial cell-like phenotype using bFGF, lean ADSCs effectively upregulate differentiation-associated miRNAs, whereas obese ADSCs exhibit a marked downregulation of this pro-angiogenic signature [56].

## 8. Effects of Massive Weight Loss on ADSC Function

Weight loss following bariatric surgery partially reverses obesity-induced alterations in ADSCs [19,23]. Mitochondrial respiration and proliferative capacity improve following substantial BMI reduction [23]. Pro-inflammatory gene expression, including *TNF-α*, *CCL5*, and *COX2*, is downregulated in ADSC monocultures after weight loss [23]. However, recovery remains incomplete across multiple parameters. Ex-obese ADSCs retain elevated secretion of the pro-inflammatory chemokine MCP-1 relative to never-obese controls, indicating incomplete normalisation of the paracrine phenotype following weight loss [19]. In macrophage co-culture, post-weight-loss ADSCs exhibit pro-inflammatory behaviour comparable to obese ADSCs, characterised by downregulation of anti-inflammatory factors and upregulation of pro-inflammatory mediators [23]. The anti-inflammatory gene *TSG6* is downregulated in weight-loss ADSC monocultures. However, in M2 macrophage co-cultures, *TSG6* expression is upregulated in weight-loss ADSCs relative to obese ADSCs, suggesting partial recovery of immunosuppressive capacity [23].

The lipid accumulation capacity of ex-obese ADSCs remains elevated relative to never-obese controls following bariatric surgery-induced weight loss [19]. This is relevant because lipid accumulation is commonly used as a readout of adipogenic differentiation. Although ex-obese ADSCs show the greatest lipid accumulation after differentiation, the predominance of perilipin-positive rather than adipose differentiation-related protein (ADRP)-positive lipid droplets suggests partial pre-commitment towards a dysfunctional mature adipocyte phenotype rather than retention of progenitor function [19]. Lower adiponectin secretion after differentiation further supports persistent adipocyte dysfunction despite comparable BMI at sampling [19]. At the structural level, ex-obese subcutaneous adipose tissue retains an altered perivascular progenitor niche, including persistent changes in perivascular ADSC subpopulations (pericyte and supra-adventitial) compared with never-obese controls [19]. Together, these findings indicate that obesity-induced alterations in ADSC behaviour, stromal composition, and vascular-associated progenitor architecture may persist despite substantial weight loss [19].

Donors in Adnan et al. [23] remained overweight or obese post-surgery, limiting the generalisability of these findings to populations achieving normal range BMI. Whether the epigenetic alterations associated with obesity in ADSCs are reversible following bariatric surgery has not been directly investigated. Peripheral blood, validated as a surrogate for epigenetic changes in adipose precursor cells, provides indirect evidence that partial rescue is possible [35]. Within 5 to 22 months post-surgery, DNA methylation at the *PANDAR* locus increased 1.25-fold, accompanied by a 33% reduction in its expression [35]. Adipose tissue has been shown to retain an epigenetic memory of obesity for at least two years following weight loss [59], consistent with the functional persistence reported at two to four years post-surgery [19,23]. Epigenetic and functional recovery following bariatric surgery appears to be a gradual, non-linear process, and certain obesity-induced alterations in ADSCs may represent a form of durable cellular scarring not fully reversed by weight loss alone [19,23,35,59]. Collectively, while bariatric surgery improves discrete metabolic and proliferative parameters, the immunomodulatory, secretory, and structural consequences of obesity in ADSCs persist after weight loss [19,23].

## 9. Implications for Research and Clinical Practice

Obesity epigenetically alters ADSCs through DNA methylation, hydroxymethylation shifts, and long non-coding RNA dysregulation, collectively driving mitochondrial dysfunction and impaired autophagy [32,33,34,35]. These changes promote premature cellular senescence, resulting in reduced stemness and impaired adipogenic differentiation [16,32,48,49]. In parallel, downregulation of key metabolic sensors shifts the ADSC secretome towards a pro-inflammatory state with diminished pro-angiogenic potential [8,40,41,45,52]. Weight loss does not appear to fully restore normal ADSC function, suggesting the presence of persistent epigenetic and functional memory [19,23,35].

In clinical practice, patient BMI is routinely used to assess surgical risk; however, metabolic health and weight history may also be critical for predicting outcomes in autologous fat grafting procedures, including treatments for skin fibrosis. This donor heterogeneity likely confounds both clinical and pre-clinical findings. ADSCs are used for their pro-angiogenic, immunomodulatory, and multilineage differentiation capacities, all of which are impaired in obesity. Therefore, future clinical trials should stratify outcomes based on donor BMI and metabolic status rather than assuming functional equivalence across ADSC populations [24]. Similarly, pre-clinical studies would benefit from better characterisation of commercially available ADSC lines, including donor BMI and comorbidities, which are often not reported.

To improve clinical predictability, rigorous in vivo studies are needed to directly compare long-term engraftment, angiogenesis, and volume retention between ADSCs derived from obese and lean donors in reconstructive settings. To our knowledge, no studies have specifically examined the effects of ADSCs or fat grafts derived from currently or previously obese patients in the treatment of skin fibrosis. Addressing this gap may help explain variability in clinical outcomes and should be a priority for future research.

Weight loss alone appears insufficient to fully restore ADSC function, even when achieved through bariatric surgery, suggesting that pharmacological interventions targeting ADSC biology may be warranted [23]. Emerging evidence supports receptor-mediated GLP-1 receptor agonist effects on ADSC function that are not wholly attributable to systemic weight reduction. In morbidly obese T2DM patients, six months of semaglutide therapy improved ADSC proliferative capacity and enhanced white and beige adipogenesis [60]. Mitochondrial metabolic function in ADSC-derived adipocytes was also improved [60]. This included upregulation of oxidative phosphorylation in white adipocytes and UCP1-driven thermogenesis in beige adipocytes [60]. These restorations occurred without statistically significant improvement in systemic insulin sensitivity, though a systemic contributory mechanism cannot be excluded [60]. Complementary clinical evidence supports a weight-loss-independent component [61]. Semaglutide produced a pro-angiogenic and anti-inflammatory ADSC-derived secretome profile that was absent in patients achieving substantially greater weight loss through bariatric surgery [61]. In vitro evidence demonstrated GLP-1R-mediated effects on obese donor-derived ADSCs [62]. Liraglutide reduced proliferation and adipogenesis whilst upregulating adiponectin [62]. These effects were fully reversed by GLP-1R antagonism, confirming a receptor-mediated mechanism [62]. Whether these benefits extend to reversal of ADSC senescence specifically remains unresolved. No published study directly measures senescence markers in this context. A prospective randomised trial incorporating p53, p21, mitochondrial biogenesis, and adipogenic lineage endpoints in ADSCs is registered but unpublished [63].

The senolytic drugs dasatinib and quercetin (D+Q) reduced adipose tissue senescent cell burden within eleven days of a three-day oral course in individuals with diabetic kidney disease [64]. Circulating SASP factors, including IL-1α, IL-6, MMP-9, and MMP-12, were concurrently reduced within eleven days of treatment completion [64]. Whether senolytic clearance of this kind can restore ADSC proliferative, differentiation-related, and secretomic function in obese individuals has not been directly examined. Obesity changes ADSC-derived EV cargo, with reductions in *miR-126*, VEGF, and MMP-2 raising questions regarding autologous EV-based therapies in obesity [17]. Whether preconditioning or donor optimisation can remediate this change or whether it translates into a change in clinical outcome warrants further investigation [65]. Whether processing methods, such as centrifugation, closed-system filtration, or differential tissue washing, can attenuate the pro-inflammatory secretome of obese-donor ADSCs remains uninvestigated; this represents a clinically relevant gap and a priority for future translational research.

Future research should establish standardised donor characterisation that extends beyond BMI to include metabolic syndrome, glycaemic control, inflammatory status, weight-loss history, and medication exposure. This is particularly important for autologous fat grafting in fibrotic skin disorders, where therapeutic efficacy may depend on ADSC-mediated immunomodulation, angiogenesis, and anti-fibrotic paracrine signalling. Prospective clinical studies should stratify currently obese, formerly obese, and never-obese donors, and correlate clinical outcomes with functional ADSC assays, including senescence burden, mitochondrial function, angiogenic capacity, immunomodulatory activity, and EV cargo. If obesity-induced dysfunction proves clinically significant, future strategies may include donor optimisation, GLP-1 receptor agonist treatment, senolytic therapy, ex vivo preconditioning, or EV engineering. This would shift ADSC-based therapy towards a model in which donor metabolic history is treated as a determinant of therapeutic potency rather than a background variable.

## Figures and Tables

**Table 1:** Key Epigenetic Changes in Obesity.

Mechanism	Epigenetic Shift	Gene/Target	Cellular Consequence	References
5mC (Stable)	Hypomethylation Hypermethylation	*TBX15* *Survivin*	Mitochondrial dysfunction Apoptotic resistance	[31,32]
5hmC (Dynamic)	Locus-specific tagging	ATP (*SLC25A4*), Autophagy (*STX12*), IL-1β	Metabolic impairment, autophagic disruption, and chronic inflammation	[27,33,34]
lncRNA (Expression)	Upregulation	*PANDAR* *HOXA11-AS1*	Premature senescence Disrupted lipid regulation	[35,37]

**Table 2:** Key mechanisms of dysfunction in ADSCs. This table summarises the major pathophysiological alterations compromising adipose stem cell function.

Mechanism	Key Findings	References
Mitochondrial dysfunction		
Structural Damage and Impaired Mitophagy	Swelling and cristae loss *TBX15* upregulation, driving mitochondrial dysfunction (in mature adipocytes)	[31,33,38]
Metabolic Shift	Depressed ATP and membrane potential Shift from glucose respiration to FAO Aberrant aerobic glycolysis and glycogen accumulation (suppresses *SIRT1/6*) Suppressed acetyl-CoA enzymes (*ACLY*, *ACSS2*)	[33,38,39,40,42]
Oxidative Failure	Elevated mitochondrial ROS and NADPH oxidase overexpression (MetS) Suppressed antioxidant enzymes (catalase, SOD3) via reduced *ACLY*/*ACSS2*	[16,38,41,42]
Premature Cellular Senescence and Decrease in Stemness		
Cellular Senescence	Upregulation of senescence markers (p16, p53, *GLB1*/SA-β-gal) Primary cilium shortening	[14,16,21,38,43]
Decrease in Stemness	Downregulation of pluripotency genes (*OCT4*, *SOX2*, *NANOG*) Transcriptomic shift toward adipocyte-like phenotype	[44,45]
Impaired Proliferation	Decline in overall proliferative capacity Diminished DNA telomere length, impaired migration and invasion	[16,46]
Autophagy and Apoptosis Dysregulation	Impaired autophagy (dysregulated *STX12*, *SLC25A4*) Survivin-mediated apoptotic resistance prevents clearance of senescent ADSCs	[27,34]
Impaired Adipogenesis		
Impaired Adipogenesis	Reduced intracellular lipid accumulation Early lineage pre-commitment	[45,48]
Transcriptional Blockade	Elevated *MAP4K4* and TNF-α suggest potential inhibition of PPAR-γ activation *ZNF521* pathway dysregulation	[21,49]
Pro-Fibrotic Reprogramming	Downregulation of adipogenic genes (*SLC2A4*, *PLIN1*) Upregulation of extracellular matrix/fibrotic proteins (*COL1A1*, *FN1*) Contributes to pericellular fibrosis and tissue rigidity	[24,46]
Pro-inflammatory Secretome		
Acquisition of SASP	Triggers elevated secretion of pro-inflammatory cytokines (TNF-α, IL-6) and chemokines (MCP-1) Decreased secretion of TGF-β1	[8,16,40]
Loss of Immunomodulation	Loss of T-cell and B-cell suppression, mechanistically linked to IL-1β signalling Failure to induce M2 macrophage polarisation CD40L-CD40 axis activation driving NF-κB-mediated IL-8 production and neutrophil recruitment	[8,51,54]
Angiogenesis		
Decreased pro-angiogenic factors Increased anti-angiogenic/inflammatory factors	Downregulation of VEGF and Adiponectin; Upregulation of *HGF*, IL-1β, and Thrombospondin-1 (TSP-1).	[19,27,28,41]
Extracellular Vesicle (EV) Depletion	Depleted pro-angiogenic factors (VEGF, MMP-2, *miR-126*).	[17]
Decreased tubulogenesis Reduced motility	Reduced intrinsic tubule formation, statistically significant in metabolic syndrome Failure to restore *VEGF* expression or stimulate tubulogenesis in injured HUVECs Impaired support of endothelial network formation in twin studies Impaired migration and invasion responses to PMA and MCP-1	[16,41,50,58]
miRNA dysregulation Loss of identity	Failure to upregulate the pro-angiogenic miRNA signature Reduced expression of the pericyte/pro-angiogenic marker CD146.	[50,56]

## Data Availability

Not applicable.

## Abbreviations

5hmC, 5-Hydroxymethylcytosine; 5mC, 5-Methylcytosine; *ACLY*, ATP-Citrate Lyase; *ACSS2*, Acyl-CoA Synthetase Short-Chain Family Member 2; ADSCs, Adipose-Derived Stem Cells; ADRP, Adipose Differentiation-Related Protein AFT, Autologous Fat Transfer; ASC, Apoptosis-Associated Speck-like Protein Containing a CARD; ATP, Adenosine Triphosphate; bFGF, Basic Fibroblast Growth Factor; BMI, Body Mass Index; *CCL5*, C-C Motif Chemokine Ligand 5; CD4, Cluster of Differentiation 4; CD11c, Cluster of Differentiation 11c; CD25, Cluster of Differentiation 25; CD29, Cluster of Differentiation 29 (Integrin Beta-1); CD40, Cluster of Differentiation 40; CD40L, CD40 Ligand; CD90, Cluster of Differentiation 90; CD106, Cluster of Differentiation 106 (VCAM-1); CD146, Cluster of Differentiation 146; *COL1A1*, Collagen Type I Alpha 1 Chain; *COX2*, Cyclooxygenase 2; D+Q, Dasatinib and Quercetin; DNA, Deoxyribonucleic Acid; ETC, Electron Transport Chain; EVs, Extracellular Vesicles; FAO, Fatty Acid Oxidation; FGF2, Fibroblast Growth Factor 2; *FN1*, Fibronectin 1; *FOXO1*, Forkhead Box O1; FOXP3, Forkhead Box P3; *GLB1*, Galactosidase Beta 1; GLP-1, Glucagon-Like Peptide-1; GLP-1R, Glucagon-Like Peptide-1 Receptor; GLP-1 RA, Glucagon-Like Peptide-1 Receptor Agonist; *HGF*, Hepatocyte Growth Factor; Hh, Hedgehog; HLA-II, Human Leukocyte Antigen Class II; *HOXA11-AS1*, HOXA11 Antisense RNA 1; HUVECs, Human Umbilical Vein Endothelial Cells; IL-1α, Interleukin-1 Alpha; IL-1β, Interleukin-1 Beta; IL-2, Interleukin-2; IL-6, Interleukin-6; IL-8, Interleukin-8; lncRNA, Long Non-Coding Ribonucleic Acid; M1, Classically Activated Macrophage; M2, Alternatively Activated Macrophage; *MAP4K4*, Mitogen-Activated Protein Kinase Kinase Kinase Kinase 4; MCD, Malonyl-CoA Decarboxylase; MCP-1, Monocyte Chemoattractant Protein-1; MetS, Metabolic Syndrome; *miR-126*, MicroRNA-126; *miR-203*, MicroRNA-203; miRNA, MicroRNA; MMP-2, Matrix Metalloproteinase-2; MMP-9, Matrix Metalloproteinase-9; MMP-12, Matrix Metalloproteinase-12; MSCs, Mesenchymal Stem/Stromal Cells; mtDNA, Mitochondrial DNA; NADPH, Nicotinamide Adenine Dinucleotide Phosphate; *NANOG*, Nanog Homeobox; NF-κB, Nuclear Factor Kappa-Light-Chain-Enhancer of Activated B Cells; *OCT4*, Octamer-Binding Transcription Factor 4; p16, Cyclin-Dependent Kinase Inhibitor 2A; p21, Cyclin-Dependent Kinase Inhibitor 1A; p53, Tumour Protein P53; *PANDAR*, Promoter of CDKN1A Antisense DNA Damage Activated RNA; PGE2, Prostaglandin E2; *PLIN1*, Perilipin 1; PMA, Phorbol 12-Myristate 13-Acetate; PPAR-γ, Peroxisome Proliferator-Activated Receptor Gamma; RNA, Ribonucleic Acid; ROS, Reactive Oxygen Species; SA-β-gal, Senescence-Associated Beta-Galactosidase; SASP, Senescence-Associated Secretory Phenotype; *SIRT1*, Sirtuin 1; *SIRT6*, Sirtuin 6; *SLC2A4*, Solute Carrier Family 2 Member 4; *SLC25A4*, Solute Carrier Family 25 Member 4; SMAD7, Mothers Against Decapentaplegic Homolog 7; *SOD3*, Superoxide Dismutase 3; *SOX2*, SRY-Box Transcription Factor 2; *STX12*, Syntaxin 12; SVF, Stromal Vascular Fraction; T2DM, Type 2 Diabetes Mellitus; *TBX15*, T-Box Transcription Factor 15; TET, Ten-Eleven Translocation; TGF-β1, Transforming Growth Factor Beta 1; *THBS1*, Thrombospondin-1; TNF-α, Tumour Necrosis Factor Alpha; *TSG6*, TNF-Stimulated Gene 6; TSP-1, Thrombospondin-1; UCP1, Uncoupling Protein 1; VCAM-1, Vascular Cell Adhesion Molecule 1; VEGF, Vascular Endothelial Growth Factor; *ZNF521*, Zinc Finger Protein 521; ΔΨm, Mitochondrial Membrane Potential.
